# Poorly Managed Stressors Contributing to an Affective Disorder in a High-Performance Athlete: A Case Report

**DOI:** 10.7759/cureus.33507

**Published:** 2023-01-08

**Authors:** Kenneth Oforeh, Terrance Tumenta, Danish Qureshi, Haroon Saeed, Stanley Nkemjika

**Affiliations:** 1 Psychiatry and Behavioral Sciences, Interfaith Medical Center, New York, USA

**Keywords:** sports psychiatry, injuries, stress, athletes, sports, psychology, psychiatry

## Abstract

Recently, there has been an increase in awareness about mental illness, especially among professional athletes. This has brought to focus the important roles that sports psychiatrists and other allied health professionals play in professional sports. It has also exposed the limitations of sports psychiatry, especially the shortage of sports psychiatrists. Professional athletes are subjected to multiple stressors during their careers, resulting in significant mental health outcomes. If not adequately addressed, stress can cause performance slumps and poorer outcomes, which in turn exacerbates stress in a vicious cycle. We present the case of a former male athlete with multiple physical, somatic, and psychosocial stressors that predisposed him to develop mental illness. This case highlights the potential consequences of a lack of early recognition and management of physical and psychosocial stressors in athletes, which can contribute to psychological illness and potentially lead to adverse medical outcomes such as repeat hospitalization and homelessness.

## Introduction

Stress could be described as a state of mental or emotional strain or tension resulting from adverse or very demanding circumstances. Evidence in the literature stipulates that chronic stress could increase the likelihood of developing mental health disorders [1]. However, the mechanism of this relationship remains unclear to date. Following research, different hypotheses have been proposed concerning the environment's role in genetic activation [2]. Notably, the release of neurochemical proteins - serotonin and adrenaline - leads to a cascade of inflammatory processes in the brain that may affect areas crucial to memory and regulating emotions [3]. Chronic stress could perpetuate prolonged activation of the immune systems leading to poor stressor homeostasis [4].

Similarly, elite athletes are not exempt from poor mental health attributable to the intense demands of top-level sports [5]. Evidence in literature has suggested the role of cumulative lifetime stress exposure, which may foster maladaptive coping strategies, increase susceptibility to future stress, and limit interpersonal relationships among athletes [6]. Additionally, studies have suggested that exposure to the intense demands of their sporting careers [7], including sport (e.g., injury, pressure to perform) and non-sport (e.g., grief, marital divorce, abuse) stressors may be partly attributable in relation to the role of the environment. These findings are more prominent in elite athletes than the non-elite athletes due to the differential professional and longer duration of the engagement [8]. Notably, elite athletes may not recognize, acknowledge, or seek support following stressors, leading to further decompensation. Despite continued research, the factors influencing elite athletes' mental health and well-being remain unclear. Thus far, there remains a paucity of literature on the role of stress in developing mental health disorders among elite athletes. Hence, we present this case report of an elite athlete with multiple chronic stressors that predisposed him to mental health disorder.

## Case presentation

The patient is a 42-year-old African American male, retired professional basketball player, single, has a daughter, currently unemployed, living in a homeless shelter, with a psychiatric history of schizoaffective disorder, bipolar type diagnosed seven years ago, and a medical history of hypertension. The patient was escorted by the police to the psychiatric emergency room because of his disorganized behavior in the streets. Upon evaluation, the patient was noted to be agitated and restless, evidenced by pacing the emergency room and being uncooperative with psychiatric emergency room protocol. He was verbally aggressive, loud, and hyper-verbose, with a lot of profane words directed at the staff. He was making inappropriate, bizarre gestures by waving his hands in the air and blinking his eyes intermittently. He appeared disorganized by singing and dancing to an unknown sound. He stated: "Look at my teeth. I'm eating pepper seeds." The patient was also noted to be putting a metal piece in his mouth, refused further searches, and became threatening toward staff, eventually requiring intramuscular psychotropic medication. He was then admitted to the inpatient psychiatric unit for safety and stabilization. The patient's onset of symptoms dates back seven years before this current presentation due to non-adherence to medications and outpatient care.

During his hospitalization, he admitted to poor sleep hygiene, stating, "I've not slept well for years since I started playing basketball professionally, and it's my usual pattern." He described his sleep as just taking random naps during flights and said he had been sleep-deprived for the past ten years due to lots of professional traveling across different time zones during regular-season competitions. The patient also stated that he had been "stressed," which he described as dealing with "physical pain and emotions" his whole life. He stated his problems started about 20 years prior when he suffered a lower back injury two years following the start of his college basketball career. After suffering the injury, he refused treatment, which worsened his back pain. Eventually, due to the worsening pain, he had surgery but was sidelined from further participation in sports for two years, during which he reported depression, fear, and anxiety owing to the uncertainty of his future. He followed up with his school physician but reported no improvement in his mood symptoms. In addition, he had poor pain management, which delayed his return to active play and led to his release. After his release, he transferred to another college, but playing time was again limited. During that time, he reported that despite worsening frustration, pain, and anger, he still had a passion for playing because basketball was the "only way out."

Subsequently, he suffered another hip injury at his new college. He underwent another surgery and was sidelined again for another six months, which ended his season prematurely. Furthermore, he reported playing professional basketball overseas two years after, and his daughter was born three years later. The patient described a problematic relationship with his daughter's mother and subsequently engaged in a custody battle that continues to date. Although the patient did not give a clear timeline of events, the patient reported that his symptoms worsened following the start of his custody battle and was subsequently referred to a general psychiatrist; however, he reported he had three hospitalizations due to non-adherence to medication and outpatient follow-up. In addition, the patient reports that he had to travel between home and abroad for court hearings during basketball competitions, which further disturbed his rest time and worsened his mood and his sleep. He denied any history of violent or threatening behavior but reported a history of intermittent anger outbursts. He denied any history of self-injurious behavior or suicide attempt, denied perceptual disturbances, denied use of any illicit substances, and denied any family psychiatric history of any mental illness. 

## Discussion

Sports psychiatrists apply the practice of psychiatry to the field of sports. They are astute at recognizing sport-specific risk factors, which are a source of stress that general psychiatrists often miss. Stress has varying concepts and definitions. According to Weiss et al. [9], stress is a "life-changing event" of varying severity, which could be positive or negative. Interestingly, our patient dealt with multiple stressors during his basketball career in the form of physical injuries, sleep disturbance, and an ongoing custody battle for his child. Considering no past psychiatric history prior to the onset of stressors, nor family history was reported in our patient, these multiple stressors could have played a part in his psychiatric presentation. Evidence in the literature supports that stress plays an essential role in developing affective disorders. In fact, Post et al., [10] in the "kindling hypothesis" postulate that the first episode of bipolar disorder is caused by significant psychological stress; however, subsequent episodes occur in a self-governed or autonomous manner. Thus, there is an increasingly discordant relationship between affective disorder's first and successive symptoms. Furthermore, stress causes the generation of pro-inflammatory cytokines such as interleukin-6, tumor necrosis factor-alpha, and interleukin-1B. These cytokines may underlie the etiopathogenesis of neurobehavioral illnesses [11].

Healthcare professionals recommend a daily sleep duration of about seven to nine or more hours, as adequate sleep is required to restore normal body physiology and energy balance [12]. In addition, certain neurotransmitters regulate and maintain the sleep-wake cycle, like dopamine, epinephrine, histamine, and serotonin. They are responsible for maintaining wakefulness in the central nervous system, while orexin facilitates their release in the thalamus [12]. These neurochemicals transmit electrical signals seen in the rapid eye movement (REM) and non-REM (NREM) stages of sleep. Each cycle typically lasts 70-120 minutes, and athletes require about four cycles, but when disrupted, they are predisposed to mental illness and performance slumps. Studies have also shown that athletes sleep poorer than the average population in terms of quality and duration [13,14]. Additionally, evidence also suggests that certain factors in sports may disrupt the mechanisms responsible for circadian rhythms and sleep switches. Some contributory factors include travel to different time zones, mental illnesses, pre-competition anxiety, use of devices with blue light, drugs, stimulants, injuries, and practice schedules [15]. These were evident in our patient, who had multiple ongoing chronic stressors that impacted his mental well-being, one of which was his sleep.

Physical injuries like lower back and hip injuries occur in basketball and other high-intensity contact sports, and there is a strong connection between physical injuries and mental health [16]. Most lower back injuries are structural (stress fractures, disc herniation, and slippage). In contrast, most hip injuries in basketball are outside the joints and are due to strains and contusions of the muscular layer, and rare occasions, stress and avulsion fractures [17]. The management typically involves a multi-disciplinary team of orthopedists, physical therapists, and chiropractors to institute rest, ice, muscle relaxants, anti-inflammatory medications, steroidal injections, rehabilitation, and other treatment modalities. If poorly managed, the direct effect of pain can be a source of stress to the mental well-being of athletes. Due to these injuries, athletes may be threatened with uncertainty with a return to play, poor sleep, poor performance, lacking confidence, muscle spasms, stiffness, and even cause early retirement [15]. Fortunately, professional basketball players have a return to play rate of about 75% following the timely multi-disciplinary intervention [18]. Sports psychiatrists skilled at monitoring sport-related stressors can quickly recognize and help with early management, including but not limited to communicating the nature and extent of illness, prescribing antidepressants, optimizing their sleep, and managing their affective response to pain. Other interventions include establishing support networks, pain control, and psychotherapy (CBT - cognitive behavioral therapy).

## Conclusions

This case highlights the potential adverse consequences of a lack of early management of stressors in an adult male basketball player, which appeared to contribute to his psychological illness, repeated hospitalization, and homelessness. In conclusion, multiple factors contribute to stress in high-intensity athletes. Chronic stress may predispose to mental illness, resulting in performance slumps and poorer outcomes. We recommend that sports teams should consider providing their athletes with access to sports psychiatrists, as to better recognize and manage sport-specific stressors.

## References

[1] Beable S, Fulcher M, Lee AC, Hamilton B (2017). SHARPSports mental Health Awareness Research Project: prevalence and risk factors of depressive symptoms and life stress in elite athletes. J Sci Med Sport.

[2] Assary E, Vincent JP, Keers R, Pluess M (2018). Gene-environment interaction and psychiatric disorders: review and future directions. Semin Cell Dev Biol.

[3] O'Mahony CM, Clarke G, Gibney S, Dinan TG, Cryan JF (2011). Strain differences in the neurochemical response to chronic restraint stress in the rat: relevance to depression. Pharmacol Biochem Behav.

[4] Hannibal KE, Bishop MD (2014). Chronic stress, cortisol dysfunction, and pain: a psychoneuroendocrine rationale for stress management in pain rehabilitation. Phys Ther.

[5] Gorczynski PF, Coyle M, Gibson K (2017). Depressive symptoms in high-performance athletes and non-athletes: a comparative meta-analysis. Br J Sports Med.

[6] Epel ES, Crosswell AD, Mayer SE, Prather AA, Slavich GM, Puterman E, Mendes WB (2018). More than a feeling: a unified view of stress measurement for population science. Front Neuroendocrinol.

[7] Souter G, Lewis R, Serrant L (2018). Men, mental health and elite sport: a narrative review. Sports Med Open.

[8] Rice SM, Purcell R, De Silva S, Mawren D, McGorry PD, Parker AG (2016). The mental health of elite athletes: a narrative systematic review. Sports Med.

[9] Weiss RB, Stange JP, Boland EM, Black SK, LaBelle DR, Abramson LY, Alloy LB (2015). Kindling of life stress in bipolar disorder: comparison of sensitization and autonomy models. J Abnorm Psychol.

[10] Post RM (1992). Transduction of psychosocial stress into the neurobiology of recurrent affective disorder. Am J Psychiatry.

[11] Benedetti F, Aggio V, Pratesi ML, Greco G, Furlan R (2020). Neuroinflammation in bipolar depression. Front Psychiatry.

[12] Watson NF, Badr MS, Belenky G (2015). Recommended amount of sleep for a healthy adult: a joint consensus statement of the American Academy of Sleep Medicine and Sleep Research Society. Sleep.

[13] Watson AM (2017). Sleep and athletic performance. Curr Sports Med Rep.

[14] Halson SL, Appaneal RN, Welvaert M, Maniar N, Drew MK (2022). Stressed and not sleeping: poor sleep and psychological stress in elite athletes prior to the Rio 2016 Olympic Games. Int J Sports Physiol Perform.

[15] Walsh NP, Halson SL, Sargent C (2020). Sleep and the athlete: narrative review and 2021 expert consensus recommendations. Br J Sports Med.

[16] Ohrnberger J, Fichera E, Sutton M (2017). The relationship between physical and mental health: a mediation analysis. Soc Sci Med.

[17] Jackson TJ, Starkey C, McElhiney D, Domb BG (2013). Epidemiology of hip injuries in the national basketball association: a 24-year overview. Orthop J Sports Med.

[18] Hoskins W, Pollard H, Daff C (2009). Low back pain status in elite and semi-elite Australian football codes: a cross-sectional survey of football (soccer), Australian rules, rugby league, rugby union and non-athletic controls. BMC Musculoskelet Disord.

